# Precocious puberty in a case of Simpson–Golabi–Behmel syndrome with a de novo 240-kb deletion including *GPC3*

**DOI:** 10.1038/s41439-022-00196-8

**Published:** 2022-06-09

**Authors:** Keisuke Watanabe, Atsuko Noguchi, Ikuko Takahashi, Mamiko Yamada, Hisato Suzuki, Toshiki Takenouchi, Kenjiro Kosaki, Tsutomu Takahashi

**Affiliations:** 1 Department of Pediatrics, Akita University Graduate School of Medicine, Akita, 010-8543 Japan; 2 Center for Medical Genetics, Keio University School of Medicine, Tokyo, 160-8582 Japan; 3 Department of Pediatrics, Keio University School of Medicine, Tokyo, 160-8582 Japan

**Keywords:** Growth disorders, Endocrine reproductive disorders

## Abstract

Here, we report a Japanese patient with Simpson–Golabi–Behmel syndrome involving a de novo 240-kb deletion including a part of *GPC3*. The patient showed pre- and postnatal macrosomia associated with coarse face, macrocephaly, supernumerary nipples, and cryptorchidism and characteristically presented with precocious puberty, mostly evaluated as advanced pubertal age of 15 years at the chronological age of 11.5 years.

Simpson–Golabi–Behmel syndrome (SGBS) is an X-linked overgrowth disorder clinically characterized by pre- and postnatal macrosomia associated with characteristic external features, including coarse face, macrocephaly, supernumerary nipples, hypospadias, cryptorchidism, and extremity abnormalities, as well as internal malformations, such as diaphragmatic hernias, cardiac defects, and gastrointestinal malformations^1–4^. SGBS is also characterized by an increased risk of malignancy development, mostly Wilms tumor and liver cancer^5^. SGBS is caused by loss-of-function mutations in the heparan sulfate proteoglycan glypican 3 gene (*GPC3*) that maps to chromosome Xq26.2^6^. *GPC3* encodes GPC3, a member of the glypican family that binds to the exocytoplasmic surface of the plasma membrane through covalent glycosylphosphatidylinositol linkage^7^. Glypicans control signaling for Hedgehog, Wnt, fibroblast growth factors, and bone morphogenic proteins^7^. GPC3 downregulates cell proliferation by suppressing Hedgehog and modulating Wnt signaling pathways^7^. Accordingly, loss-of-function of GPC3 causes hyperactivation of Hedgehog signaling, which possibly correlates with the overgrowth and increased tumor risk in SGBS.

To date, more than 80 mutations responsible for SGBS have been identified^7^. In analyses of 120 unrelated patients with SGBS, these mutations were classified into eight groups: large deletions (34.9%), frameshift mutations leading to a premature stop codon (24.4%), nonsense mutations (16.3%), missense mutations (8.1%), large duplications (8.1%), splice site mutations (4.7%), translocations (2.3%), and frameshift mutations (1.2%). In this report, we describe a unique de novo 240-kb gene deletion including a part of *GPC3* and an interesting endocrinological complication of precocious puberty and advanced bone age in a Japanese patient with SGBS.

A 14.5-year-old boy presented with intellectual disability, overgrowth, macrocephaly, dysmorphic facial features, and supernumerary nipples (Fig. 1a, b, c).

**Fig. 1:**
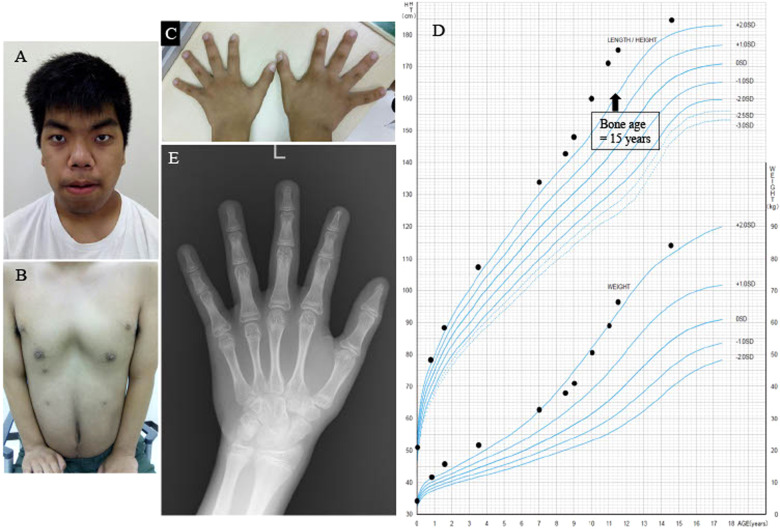
Images of the face, supernumerary nipples, hands, growth curve, and radiography of the left hand. **a** The patient exhibits macrocephaly. **b** The patient shows bilateral supernumerary nipples. **c** The patient’s hands show hypoplastic nails. **d** Growth curve showing accelerated growth velocity around the age of 10 because of early onset of puberty. **e** A radiograph of the left hand shows a bone age of 15 years at the chronological age of 11.5 years. Written informed consent was obtained from the patient’s family for publication of this clinical report and accompanying images.

The patient was born to Japanese nonconsanguineous parents, with a birth weight of 4079 g (+2.0 SD), height of 51.0 cm (+1.0 SD), and head circumference of 36.0 cm (+2.8 SD) at the gestational age of 37 weeks and 2 days. During the pregnancy, amniocentesis was performed four times for amniotic fluid decompression because of polyhydramnios, and the combined fetal chromosome test showed a normal karyotype at the gestational age of 22 weeks.

After birth, the neonate was suspected to have a congenital anomaly disease because of his peculiar face and prenatal overgrowth, macrocephaly, and bilateral undescended testes. Laboratory tests, including urinary uronic acid tests for mucopolysaccharidoses, failed to diagnose the patient.

During his early childhood, the patient grew to a tall stature: his height was more than +2.0 SD of the Japanese standard growth (Fig. 1d). At the age of 11.5 years, the patient showed overgrowth and advanced pubertal onset, with a height and weight of 175 cm (+4.0 SD) and 67.0 kg (+2.1 SD), respectively. The patient was then endocrinologically evaluated as presenting advanced pubertal onset owing to pubertal levels of luteinizing hormone (0.8 mIU/mL), follicular stimulating hormone (2.1 mIU/mL), and insulin-like growth factor-1 (511 ng/mL). His testicular volume was 25 mL bilaterally, which was comparable to that at 15 years. His bone age was advanced and was assessed as being equivalent to that of a 15-year-old boy, leading to the diagnosis of precocious puberty (Fig. 1e). At the age of 14.5 years, he was 184.5 cm tall (+2.4 SD) and weighed 84.0 kg (+2.1 SD), which was evaluated as overgrowth, compared to the height of his parents, which were 174.5 cm for his father and 154 cm for his mother.

We suspected congenital overgrowth syndromes, including SGBS, in the diagnosis of this patient. To identify causal variants, we performed whole-genome sequencing of both the patient and his parents. Ethical approval for this study was obtained from the Ethics Committee of Akita University Graduate School of Medicine, Akita, Japan. Blood samples were collected after obtaining written informed consent from the parents. Whole-genome sequencing was performed on samples using a short-read sequencer (NovaSeq6000; Illumina) according to the manufacturer’s instructions. The samples were processed through an alignment and structural variant detection pipeline using the DRAGEN 3.5 suite for Illumina data. The presence of a 240-kb deletion (ChrX: 132,624,991–132,865,393) (GRCh37/hg19) including exons of *GPC3* was detected in the patient but not in the parents (Fig. 2).

**Fig. 2:**
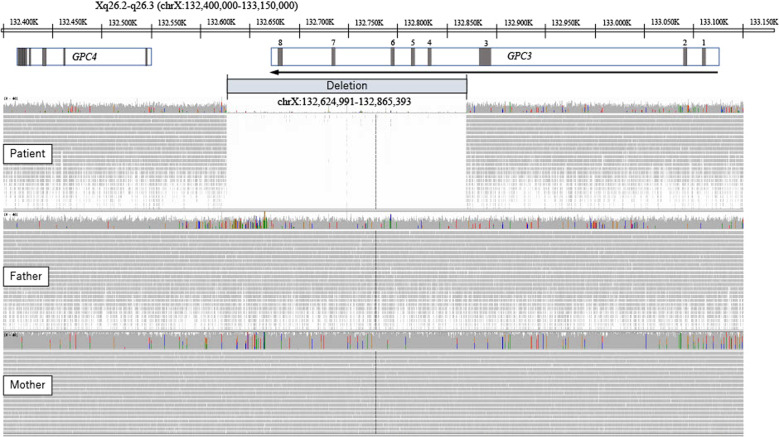
Gene deletion in the patient. A de novo gene deletion, chrX: 132,624,991_132,865,393, was found in the patient (pt) but not in the father (fa) or mother (mo). The arrow indicates the direction of *GPC3*. The gray part in the box of *GPC3* indicates each exon.

There have been no reports of endocrinological complications or descriptions of a pubertal state in patients with SGBS. Indeed, this is the first SGBS case clinically described as complicated by precocious puberty or early-onset pubertal progression, resulting in a tall adult height of above +2.0 SD.

Bone age has been evaluated in nine unrelated cases of SGBS, though there are no reports of accompanying pubertal complications. During early childhood from birth to 8 years of age, five of eight children with SGBS showed advanced bone age^8–11^, suggesting prenatal or postnatal accelerated growth velocity in SGBS. During middle childhood, namely, from 8 to 12 years of age, a 10.4-year-old boy was evaluated as having a bone age of a 12.5-year-old^11^; however, the pubertal stage for this patient was not described, suggesting a lack of relationship between advanced bone age and advanced pubertal progression. In our case of this 11.5-year-old boy, bone age was evaluated to be that of a 15.0-year-old, which was advanced by 3.5 years, presumably due to the early onset of puberty. A diagnosis of precocious puberty was suggested due to pubertal levels of luteinizing hormone and follicle-stimulating hormone and pubertal testicular volumes of 15 mL bilaterally.

Among the group of overgrowth syndromes, including Sotos syndrome, Beckwith–Wiedemann spectrum, and Weaver syndrome, Sotos syndrome is known to result in advanced bone age in prepubertal subjects^12,13^. Precocious puberty has also been observed in three unrelated patients with Sotos syndrome^14–16^. For Beckwith–Wiedemann spectrum, advanced bone age was reported as a characteristic finding, particularly during the first 4 years after birth^17^. Weaver syndrome has also been described as an overgrowth syndrome associated with advanced bone age^18,19^. Nevertheless, to the best of our knowledge, there have been no reports of precocious puberty in either Beckwith–Wiedemann syndrome or Weaver syndrome. Our report suggests a clinical similarity in the endocrinological aspects between Sotos syndrome and SGBS.

## Data Availability

The relevant data from this Data Report are hosted at the Human Genome Variation Database at.10.6084/m9.figshare.hgv.3196.
